# Cannabinoid CB1 Receptor Activation Mediates the Opposing Effects of Amphetamine on Impulsive Action and Impulsive Choice

**DOI:** 10.1371/journal.pone.0025856

**Published:** 2011-10-07

**Authors:** Joost Wiskerke, Nicky Stoop, Dustin Schetters, Anton N. M. Schoffelmeer, Tommy Pattij

**Affiliations:** Department of Anatomy and Neurosciences, VU University Medical Center, Amsterdam, The Netherlands; Chiba University Center for Forensic Mental Health, Japan

## Abstract

It is well known that acute challenges with psychostimulants such as amphetamine affect impulsive behavior. We here studied the pharmacology underlying the effects of amphetamine in two rat models of impulsivity, the 5-choice serial reaction time task (5-CSRTT) and the delayed reward task (DRT), providing measures of inhibitory control, an aspect of impulsive action, and impulsive choice, respectively. We focused on the role of cannabinoid CB1 receptor activation in amphetamine-induced impulsivity as there is evidence that acute challenges with psychostimulants activate the endogenous cannabinoid system, and CB1 receptor activity modulates impulsivity in both rodents and humans. Results showed that pretreatment with either the CB1 receptor antagonist/inverse agonist SR141716A or the neutral CB1 receptor antagonist O-2050 dose-dependently improved baseline inhibitory control in the 5-CSRTT. Moreover, both compounds similarly attenuated amphetamine-induced inhibitory control deficits, suggesting that CB1 receptor activation by endogenously released cannabinoids mediates this aspect of impulsive action. Direct CB1 receptor activation by Δ9-Tetrahydrocannabinol (Δ9-THC) did, however, not affect inhibitory control. Although neither SR141716A nor O-2050 affected baseline impulsive choice in the DRT, both ligands completely prevented amphetamine-induced reductions in impulsive decision making, indicating that CB1 receptor activity may decrease this form of impulsivity. Indeed, acute Δ9-THC was found to reduce impulsive choice in a CB1 receptor-dependent way. Together, these results indicate an important, though complex role for cannabinoid CB1 receptor activity in the regulation of impulsive action and impulsive choice as well as the opposite effects amphetamine has on both forms of impulsive behavior.

## Introduction

Impulsivity is a multifaceted construct covering various, largely independent, behavioral measures ranging from impulsive actions, e.g. disturbed inhibitory control and response inhibition, to impulsive decisions, e.g. delay aversion [1]–[4]. Maladaptive impulsivity has been implicated in a wide range of psychiatric and neurological disorders, including Attention–Deficit/ Hyperactivity Disorder (ADHD), bipolar disorder, Parkinson's disease, and substance use-related disorders [5]. Unraveling the neurobiology of impulsivity may allow the development of novel pharmacotherapies to treat maladaptive impulsivity and is therefore of utmost importance.

Traditionally, studies on impulsivity have primarily focused on the role of monoamine neurotransmission [4], [6]. Interestingly, other neurotransmitters have also been implicated in impulsivity, including endogenous cannabinoids [3]. The endogenous cannabinoid system, named after the fact that it is activated by Δ9-Tetrahydrocannabinol (Δ9-THC), the principle active component of herbal *cannabis sativa*, includes at least two G-protein coupled receptors, CB1 and CB2 receptors, and several endogenous ligands including N-arachidonoyl-ethanolamide (anandamide) and 2-arachidonyl glycerolanandamide (2-AG) [7]–[9]. CB1 receptors are the predominant cannabinoid receptors in the central nervous system with a particular abundance in brain regions comprising the mesocorticolimbic system [10], [11]. In the brain the endogenous cannabinoid system functions to modulate synaptic activity by controlling release of virtually all other neurotransmitters, including GABA, glutamate, and dopamine (DA) [12]. Considering its abundance and cellular function in the brain, it is not surprising that the CB1 receptor has been implicated in regulating many different behaviors, including higher-order cognitive or executive functions such as attentional processing, behavioral flexibility, and impulsivity [13]–[15]. With respect to the latter, it has been shown that both chronic and acute use of Δ9-THC can affect impulsive behavior in humans [16]–[19]. Moreover, two recent preclinical studies found evidence for a role for CB1 receptors in modulating specific aspects of impulsivity, as it was found that the CB1 receptor antagonists/inverse agonists SR141716A and SLV330 increased inhibitory control in rats [20], [21]. In addition, it is noteworthy that polymorphisms in the CB1 receptor gene (CNR1 gene) have been linked to impulsivity [22] and the development of ADHD [23], [24], and that ADHD patients were recently found to have decreased anandamide degradation as compared to healthy control subjects [25].

Currently, the most widely prescribed drugs to treat ADHD and maladaptive impulsivity are the psychostimulants methylphenidate and amphetamine, which enhance monoamine neurotransmission [26], [27]. Somewhat paradoxically, acute challenges with amphetamine decrease inhibitory control in humans and rodents, i.e. increase impulsive action, at least when operationalized as the inability to restrain inappropriate behavior [28]–[34], while reducing impulsive choice, measured as an intolerance to delayed gratification or delay aversion [34]–[39]. These opposite effects of amphetamine are well-known to depend on enhanced DA transmission [28], [29], [31]–[33], [36], [38], [39]. Nonetheless, interactions with other neurotransmitter systems including the endogenous opioid and 5-HT systems [34], [38], [39] have also been implicated. Recent studies showed that psychostimulants such as amphetamine can acutely affect forebrain endocannabinoid levels [40]–[42], although these data remain rather inconclusive considering the exact direction of the effects. Moreover, CB1 receptor activity has been shown to modulate amphetamine-induced behavioral responses [42]–[47]. This raises the question whether CB1 receptor activation is involved in the mechanism of action, and possibly clinical effects, of amphetamine on impulsive behavior. Collectively, these observations do suggest a role for CB1 receptors in (amphetamine-induced) impulsivity, thereby highlighting the endocannabinoid system as a potential target for novel anti-impulsivity pharmacotherapies. To further elucidate this, we here investigated the role of CB1 receptors in the effects of amphetamine on impulsive action and impulsive choice, two behaviorally, neuroanatomically and neurochemically distinct forms of impulsivity [1]. Specifically, to this aim we employed systemic drug injections to manipulate impulsive behavior in two widely employed models of impulsivity, namely the 5-choice serial reaction time task (5-CSRTT) and delayed reward task (DRT), measuring impulsive action and impulsive choice, respectively [4]. Results show an important role for cannabinoid CB1 receptor activity in the regulation of impulsive action and impulsive choice as well as the opposite effects amphetamine has on both forms of impulsive behavior, although questions about the underlying mechanism(s) remain.

## Materials and Methods

### Subjects

Male Wistar rats were obtained from Harlan CPB (Horst, The Netherlands). At the start of the experiments animals weighed approximately 250 grams, and were housed two per cage in macrolon cages (42.5×26.6×18.5 cm; length×width×height) under a reversed 12 hr light/dark cycle (lights on at 7.00 p.m.) at controlled room temperature (21±2°C) and relative humidity of 60±15%. Animals were maintained at approximately 90% of their free-feeding weight, starting one week prior to the beginning of the experiments by restricting the amount of standard rodent food pellets (Harlan Teklad Global Diet, Blackthorn, UK). Water was available ad libitum throughout the entire experiment. All experiments were conducted with the approval of the animal ethical committee of the VU University Amsterdam, the Netherlands (protocol numbers: MFal04-08, ANW08-04, ANW08-05), and all efforts were made to minimize animal suffering.

### Drugs

SR141716A and Δ9-tetrahydrocannabinol (Δ9-THC) were generated and kindly provided by respectively Abbott and Echo Pharmaceuticals B.V. (both Weesp, the Netherlands). (+)-Amphetamine sulfate (OPG, Utrecht, the Netherlands) was dissolved in sterile saline, whereas SR141716A, O-2050 (Tocris Bioscience, Bristol, UK), and Δ9-THC were dissolved in a mixture of ethanol, Tween80, and sterile saline (ratio 1∶1∶18) as described before [48]. Drug doses and injection times were based on previous studies [21], [32], [36], [49], [50]. SR141716A and O-2050 were injected 45 min prior to testing, Δ9-THC 30 min prior to testing, and amphetamine 20 min prior to testing. Drugs were freshly prepared on each test day and injected intraperitoneally (i.p.) in a volume of 1 ml/kg bodyweight according to a Latin square within-subjects design. Drug tests were conducted on Tuesdays and Fridays with baseline training sessions on the other weekdays. Prior to the first test day, all animals had been habituated to i.p. saline injections twice.

### Apparatus

Experiments were conducted in identical rat five hole nose poke operant chambers with stainless steel grid floors (MED-NPW-5L, Med Associates Inc., St. Albans, VT, USA) housed in sound-insulating and ventilated cubicles. Set in the curved wall of each box was an array of five holes. Each nose poke unit was equipped with an infrared detector and a yellow light emitting diode (LED) stimulus light. Rodent food pellets (45 mg, Formula P, Bio-Serv, Frenchtown, USA) could be delivered at the opposite wall via a dispenser. In addition, a white house light could illuminate the chamber. A computer equipped with MED-PC version 1.17 (Med Associates Inc.) controlled experimental sessions and recorded data. Animals were tested once daily from Monday to Friday, during the dark phase of the light/dark cycle.

### Behavioral procedures

Separate groups of animals (*n* = 14) were trained for each experiment involving a different drug (combination) and/or task, unless stated otherwise. Specifically, in total four groups of rats were used for the experiments involving the 5-CSRTT: one group for the tests involving SR141716A in combination with amphetamine and the tests with SR141716A in combination with lengthened intertrial interval (ITI), a second group for the dose-response curve with O-2050, a third group for the tests involving O-2050 in combination with amphetamine and the tests with O-2050 in combination with lengthened ITI, and a final group for the the tests involving Δ9-THC and the tests with Δ9-THC in combination with lengthened intertrial interval (ITI). For the experiments involving the DRT, three groups of rats were used: one group for the experiment involving SR141716A in combination with amphetamine, a second group for the experiment involving O-2050, and a third group for the dose-response curve with Δ9-THC as well as the experiment involving the combination of Δ9-THC and SR141716A. For both paradigms similar habituation and magazine training protocols were followed. This protocol consisted of a habituation exposure to the operant chambers for 20 min. with the house light on and the food cup containing three food pellets during the first session. Subsequently, in the next two sessions, in total 75 pellets were delivered with an average delay of 15 seconds (s) to allow the animals to associate the sound of pellet delivery with reward.

### 5-Choice Serial Reaction Time Task

A detailed description of the 5-CSRTT behavioral procedure in our laboratory has been provided previously [32]. In short, rats were trained to detect and respond to a brief visual stimulus in one of 5 nose poke units in order to obtain a food reward. Each session terminated after 100 trials or 30 min, whichever occurred first. Initially the duration of this stimulus was 32 s and was gradually decreased to 1 s over sessions until animals reached stable baseline performance (accuracy >80% correct choice and <20% errors of omission). Responding during stimulus presentation or within the limited hold (LH) period of 2 s was counted as a correct response. Incorrect responses, premature responses during the fixed 5 s ITI, and errors of omission (no responses or a response after the LH) did not lead to the delivery of a food reward and resulted in a 5 s time-out period during which the houselight was extinguished. Importantly, when drug effects were studied under conditions with lengthened ITI duration, only for those specific test days, a fixed 7 s ITI was used. Perseverative responses after correct choice i.e. repeated responding during stimulus presentation into any stimulus unit following correct stimulus detection and before pellet collection, were measured but did not have any programmed consequences. The number of premature responses was used as an index for inhibitory control. In addition, the following other behavioral parameters were measured that reflect task performance: 1) accurate choice, i.e. percentage correct responses calculated as [number correct trials/(correct + incorrect trials)]*100; 2) omission errors, i.e. the total number of omitted trials during a session; 3) the total number of perseverative responses after correct choice, measuring aspects of compulsive behavior [51]; 4) latency to make a correct choice, i.e. the mean time between stimulus onset and nose poke in the illuminated unit; and 5) feeder latency, i.e. the latency to collect a pellet following correct choice.

### Delayed Reward Paradigm

The delayed reward paradigm used in our laboratory has been described previously [36]. Briefly, in the final stages of training and during drug testing, a session was divided into 5 blocks of 12 trials, each block started with 2 forced choice trials. Each rat received a left forced and a right forced trial, in random order. In the next 10 trials, the animals had a free choice and both the left and right units were illuminated. Poking into one position resulted in the immediate delivery of a small reinforcer (1 food pellet), whereas a nose poke into the other position resulted in the delivery of a large, but delayed, reinforcer (4 food pellets). If an animal did not make a response during this choice phase within 10 s, an intertrial interval was initiated and the trial was counted as an omission. The position associated with the small and large reinforcer was always the same for each individual, and counterbalanced for the group of rats. Delays for the large reinforcer progressively increased within a session per block of 12 trials as follows: 0, 5, 10, 20 and 40 s. Responding into non-illuminated units during the test was recorded, but had no further programmed consequences. The behavioral measure to assess task performance, i.e. the percentage preference for the large reinforcer as a function of delay, was calculated as the number of choices for the large reinforcer/(number choices large + small reinforcers) *100. Furthermore, we calculated the total number of omitted choice trials per block of 10 choice trials within a session.

### Statistical analyses

All data were analyzed using NCSS2007 version 07.1.18 (NCSS, LLC., Kaysville, UT, USA). Data were subjected to repeated measures analyses of variance (ANOVAs) with drug treatment (5-CSRTT, DRT) and delay to large reinforcer (DRT) as within subjects variables, except for 5-CSRTT experiments involving lengthened ITI duration. In that case, data were subjected to two-way ANOVAs with drug treatment and ITI duration as within subjects variables. Additional two-way repeated measures ANOVAs were performed for the 5-CSRTT experiments involving co-administration of SR141716A/O-2050 and amphetamine, to test for drug-interaction effects on premature responding as all compounds affected this behavioral measure. When appropriate, homogeneity of variance across groups was determined using Mauchly's tests for equal variances and in case of violation of homogeneity, Huynh-Feldt epsilon (ε) adjusted degrees of freedom were applied and the resulting more conservative probability values depicted and used for subsequent analyses. In case of statistically significant main effects, further post-hoc comparisons were conducted using Newman-Keuls multiple comparison tests. The level of probability for statistically significant effects was set at 0.05. All graphs were produced using GraphPad Prism version 5.02 for Windows (GraphPad Software, San Diego, CA, USA).

## Results

### Effects of SR141716A on amphetamine-induced impulsivity

**Figure 1:**
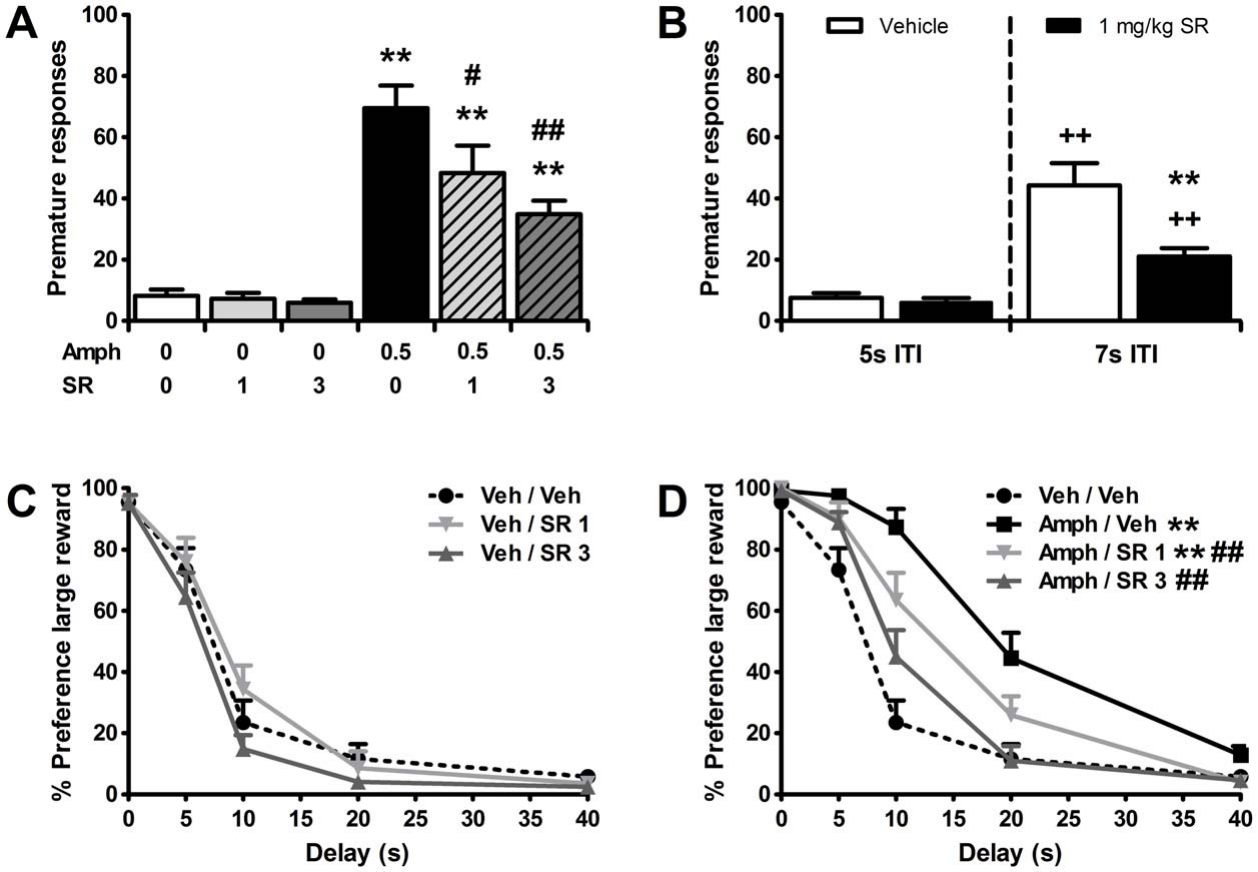
Pretreatment with the CB1 receptor antagonist/inverse agonist SR141716A attenuates amphetamine-induced impulsive behaviors. Effects of acute administration of 0.5 mg/kg amphetamine (AMPH), SR141716A (SR), and their combination on the mean (± SEM) number of premature responses made in the 5-CSRTT (a,b) and percentage preference for the larger, delayed reinforcer in the DRT (c,d). ITI: intertrial interval. In total *n* = 13-14 animals were included in the analyses. Drug doses are expressed as mg/kg. ^**^ *p*<0.005 versus respective Vehicle or Vehicle-Vehicle control; ^#^ *p*<0.05 and ^##^ *p*<0.005 compared to Amphetamine-Vehicle, ^++^p<0.005 vs respective ITI 5 s control.

As a first attempt to test the putative involvement of the endogenous cannabinoid system in amphetamine-induced impulsivity, the effects of amphetamine alone and in combination with the selective CB1 receptor antagonist/inverse agonist SR141716A (*in vitro* Ki ∼1.8 and 514 nM for CB1 and CB2 receptors, respectively [52]) were studied, first in the 5-CSRTT. One animal was excluded from the analyses due to consistent high omission rates during baseline training and drug testing (>35 omissions/session). In line with previous reports [28], [29], [31], [32], [34], a systemic injection of amphetamine (0.5 mg/kg) significantly increased premature responding in the 5-CSRTT (Fig. 1a) and prior administration of SR141716A dose-dependently attenuated this effect (*F*_5,60_ = 11.57, *p*<0.001). As summarized in Table 1, significant treatment effects were also observed on accurate choice (*F*_5,60_ = 11.57, *p*<0.001), with amphetamine reducing the percentage of correct choices and SR141716A dose-dependently reversing this deteriorating effect. Amphetamine also decreased correct response latencies (*F*_5,60_ = 7.55, *p*<0.001), and this effect was also prevented by prior treatment with SR141716A. No significant effects of any treatment combination were observed for the number of omission errors (*F*_5,60_ = 1.42, NS, ε = 0.58), perseverative responses (*F*_5,60_ = 2.66, *p*<0.1, ε = 0.39) or feeder latency (*F*_5,60_ = 2.15, *p* = 0.1, ε = 0.69).

**Table 1:** Effects of 0.5 mg/kg amphetamine (AMPH), the CB1 receptor antagonist/inverse agonist SR141716A (SR) and their combination on measures of attentional function, compulsivity, and motivation in the 5-CSRTT.

Treatment (mg/kg)	Accuracy (%)	Perseverative responses	Correct response latency (ms)	Omissions	Feeder latency (ms)
**Vehicle–Vehicle<\emph>**	94.6±0.9	6.8±1.6	598±22	10.5±3.1	1632±106
**Vehicle–SR 1<\emph>**	94.8±0.8	7.0±1.0	627±34	12.8±4.0	1727±137
**Vehicle–SR 3<\emph>**	93.0±1.5	6.4±1.1	620±31	13.2±3.6	2038±365
**AMPH–Vehicle<\emph>**	86.2±1.4**	6.4±1.4	534±21*	8.5±2.5	1441±101
**AMPH–SR 1<\emph>**	88.7±1.0**	15.6±5.4	564±25	7.6±1.6	2139±342
**AMPH–SR 3<\emph>**	89.8±1.3** #	12.2±2.4	577±20	9.1±1.4	2168±359

In total n = 13 animals were included in the analyses and data depict mean±SEM.

*p<0.05.

**p<0.005 versus Vehicle-Vehicle.

# *p*<0.05 compared to AMPH-Vehicle.

Importantly, in contrast with previous data [21], SR141716A (1 and 3 mg/kg) by itself did not significantly increase inhibitory control. However, this discrepancy probably reflected a floor effect in the current experiment. In the same group of animals, but tested over four additional test days, 1 mg/kg SR141716A did significantly reduce the number of premature responses when the duration of the intertrial interval (ITI) on test days was increased from 5 s to 7 s (Fig. 1b.; Treatment: *F*_1,13_ = 14.98, *p* = 0.002; ITI: *F*_1,13_ = 42.02, *p*<0.001; Treatment×ITI: *F*_1,13_ = 11.85, *p* = 0.004), a procedural manipulation known to robustly increase impulsivity in the 5-CSRTT in a baseline (5 s ITI)-dependent way [53], [54]. As shown in Table 2, there further was a significant treatment effect on the number of omissions made (Treatment: *F*_1,13_ = 5.57, *p* = 0.035; ITI: *F*_1,13_ = 0.27, NS; Treatment×ITI: *F*_1,13_ = 1.47, NS), with 1 mg/kg SR141716A significantly increasing the omission rate. In addition, significant effects of lengthening the ITI duration were found on correct response latencies (Treatment: *F*_1,13_ = 3.01, NS; ITI: *F*_1,13_ = 37.88, *p*<0.001; Treatment×ITI: *F*_1,13_ = 1.62, NS) and feeder latencies (Treatment: *F*_1,13_ = 1.49, NS; ITI: *F*_1,13_ = 10.12, *p* = 0.007; Treatment×ITI: *F*_1,13_ = 0.00, NS), with both types of latencies being shortened by lengthening the ITI duration, independent of prior drug treatment. No significant effects of treatment or ITI were found on accurate choice (Treatment: *F*_1,13_ = 1.09, NS; ITI: *F*_1,13_ = 3.27, *p*<0.1; Treatment×ITI: *F*_1,13_ = 0.96, NS) and perseverative responding (Treatment: *F*_1,13_ = 1.03, NS; ITI: *F*_1,13_ = 0.10, NS; Treatment×ITI: *F*_1,13_ = 0.46, NS). To control for possible effects of SR141716A alone on premature responding that remained undetected in the initial analyses and might hamper the interpretation of the amphetamine + SR141716A experiment, the dataset was also analyzed using a two-way repeated measures ANOVA. Results showed significant overall effects for both amphetamine (*F*_1,12_ = 113.65, *p*<0.001) and SR141716A (*F*_2,24_ = 6.04, *p* = 0.007) administration as well as an interaction effect between the two treatments (*F*_2,24_ = 4.98, *p* = 0.016), suggesting that the effects of SR141716A on attenuating premature responding were larger in the presence of amphetamine compared to its own effect on this parameter.

**Table 2:** Effects of the CB1 receptor antagonist/inverse agonist SR141716A (SR) on measures of attentional function, compulsivity, and motivation in the 5-CSRTT under conditions of normal or lengthened intertrial interval (ITI).

Treatment (mg/kg)	ITI (s)	Accuracy (%)	Perseverative responses	Correct response latency (ms)	Omissions	Feeder latency (ms)
**Vehicle<\emph>**	5	92.6±1.0	7.2±1.0	643±24	11.5±2.5	1760±140
**SR 1<\emph>**	5	92.7±1.3	7.4±1.2	656±24	16.5±3.2*	1861±163
**Vehicle<\emph>**	7	89.1±1.4	5.9±2.2	591±19++	11.9±2.4	1469±95+
**SR 1<\emph>**	7	91.3±1.6	7.7±2.1	624±25++	13.3±2.9*	1580±93+

In total n = 14 animals were included in the analyses and data depict mean±SEM.

*p<0.05 compared to respective Vehicle control.

+ p<0.05.

++ p<0.005 compared to respective ITI = 5 s control.

Since impulsivity is thought to be of a multifaceted nature covering various behavioral measures with only partially overlapping underlying mechanisms [1]–[4], [55], we next aimed to determine whether our results in the 5-CSRTT would generalize to another modality of impulsive behavior, namely impulsive choice. To that end, the effects of amphetamine alone and in combination with SR141716A were studied in the DRT. One animal was removed from the analysis due to a high number of omissions (>50% of choice trials in each delay block) under vehicle conditions. As previously observed [34], [36]–[39], amphetamine in this task reduced impulsive choice as reflected by an increased preference for the larger reinforcer over increasing delays as compared to vehicle treatment (Treatment: *F*_5,60_ = 17.30, *p*<0.001; Treatment×Delay: *F*_20,240_ = 6.21, *p*<0.001, ε = 0.63). Comparable to the 5-CSRTT findings, prior administration of SR141716A dose-dependently antagonized the effects of amphetamine in the DRT, without altering impulsive choice by itself (Fig. 1c,d). Together, these results indicate that CB1 receptor activity modulates amphetamine-induced impulsivity in the 5-CSRTT (impulsive action) as well as the DRT (impulsive choice).

### Effects of O-2050 on amphetamine-induced impulsivity

**Figure 2:**
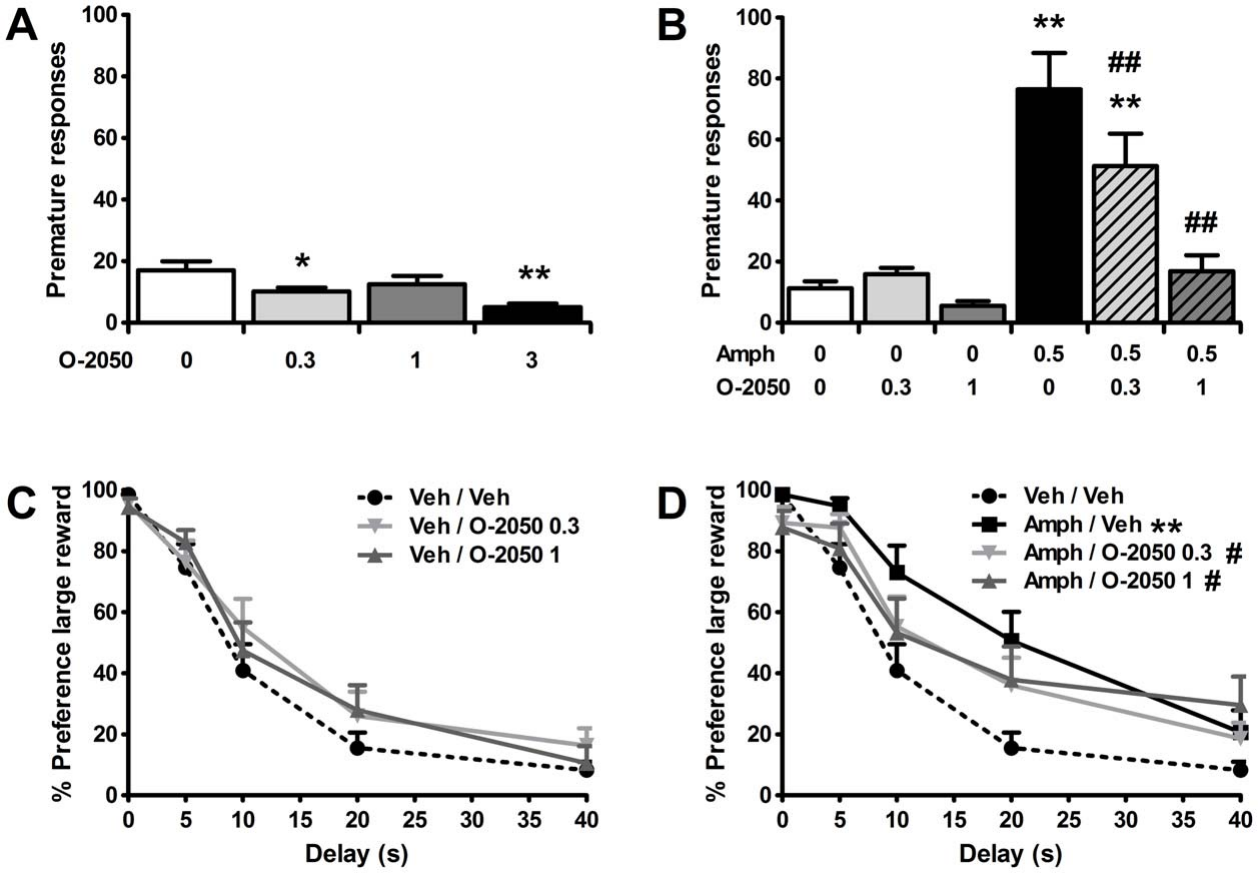
Pretreatment with the neutral CB1 receptor antagonist O-2050 also attenuates amphetamine-induced impulsive behaviors. Effects of acute administration of 0.5 mg/kg amphetamine (AMPH), O-2050, and their combination on the mean (± SEM) number of premature responses made in the 5-CSRTT (a,b) and percentage preference for the larger, delayed reinforcer in the DRT (c,d). In total *n* = 13−14 animals were included in the analyses. Drug doses are expressed as mg/kg. ^*^p<0.05 and ^**^ *p*<0.005 versus Vehicle or Vehicle-Vehicle; ^#^ *p*<0.05 and ^##^ *p*<0.005 compared to Amphetamine-Vehicle.

A possible confounding factor of employing SR141716A is this ligand's potential inverse agonistic action at CB1 receptors [56]. To exclude that the observed effects of SR141716A on impulsive behavior were due to its inverse agonistic properties, and to test the effects of a structurally unrelated CB1 receptor antagonist, two separate groups of rats were trained in the 5-CSRTT to test the effects of O-2050, a neutral, non-selective CB1 receptor antagonist (*in vitro* Ki ∼2.5 and 0.2 nM for CB1 and CB2 receptors, respectively [57]) lacking inverse agonistic properties [49], [57], [58], alone and in combination with amphetamine. By itself, similar to the previously reported effects of SR141716A [21], O-2050 dose-dependently increased inhibitory control (Fig. 2a; *F*_3,39_ = 15.82, *p*<0.001) and accurate choice (Table 3; *F*_3,39_ = 4.61, *p* = 0.007). Furthermore, at the highest dose (3 mg/kg), this neutral CB1 receptor antagonist increased the number of omissions (*F*_3,39_ = 15.82, *p*<0.001) and correct response latencies (*F*_3,39_ = 19.50, *p*<0.001). Finally, a significant overall effect was observed for feeder latencies (*F*_3,39_ = 6.97, *p*<0.001), although further post-hoc tests revealed no significant dose effects compared to vehicle administration.

**Table 3:** Effects of 0.5 mg/kg amphetamine (AMPH), the neutral CB1 antagonist O-2050, and their combinations on measures of attentional function, compulsivity, and motivation in the 5-CSRTT.

Treatment (mg/kg)	Accuracy (%)	Perseverative responses	Correct response latency (ms)	Omissions	Feeder latency (ms)
**O-2050 alone**					
**Vehicle**	85.6±1.5	8.6±2.6	587±16	5.9±0.8	1348±83
**O-2050 0.3**	89.8±1.3*	7.6±2.1	599±19	6.0±1.0	1360±73
**O-2050 1**	89.4±1.6	7.1±1.8	611±24	13.8±3.8	1620±155
**O-2050 3**	91.5±2.0**	5.1±1.0	760±24**	32.4±5.7**	1766±122
**Amphetamine+O-2050**					
**Vehicle–Vehicle**	82.3±1.5	7.1±1.6	645±32	12.2±2.3	2059±292
**Vehicle–O-2050 0.3**	84.3±1.7	9.1±2.1	672±32	14.5±2.9	1916±193
**Vehicle–O-2050 1**	88.6±1.8*	7.5±1.8	790±41*	23.1±3.8*	2115±374
**AMPH–Vehicle**	74.7±1.8*	5.3±2.3	615±19	9.5±1.8	1732±229
**AMPH–O-2050 0.3**	79.4±3.0#	4.0±1.1	619±19	11.8±3.1	1664±236
**AMPH–O-2050 1**	82.0±2.8##	6.6±2.2	701±24#	38.3±5.7** ##	4446±1642

Respectively, n = 14 and n = 13 animals were included in the analyses for O-2050 alone and AMPH+O-2050, and data depict mean±SEM.

*p<0.05.

**p<0.005 versus Vehicle or Vehicle-Vehicle.

# *p*<0.05.

## *p*<0.005 compared to AMPH-Vehicle.

For the experiment with amphetamine and O-2050, one rat needed to be excluded as data on one of the test days was missing for this animal due to technical problems. Similar to SR141716A, pretreatment with O-2050 dose-dependently antagonized amphetamine-induced increments in premature responding in the 5-CSRTT (Fig. 2b; *F*_5,60_ = 21.85, *p*<0.001, ε = 0.61). Moreover, similar to the amphetamine+SR141716A experiment, the lack of effect of O-2050 on inhibitory control under baseline (without amphetamine) conditions probably reflected a floor effect, as 0.3 mg/kg O-2050 in the same animals was able to reduce premature responding when in separate test sessions the ITI duration was increased to 7 s (data not shown; Treatment: *F*_1,13_ = 7.11, *p* = 0.02; ITI: *F*_1,13_ = 162.15, *p*<0.001; Treatment×ITI: *F*_1,13_ = 7.70, *p* = 0.02). As in the amphetamine+SR141716A experiment, additional two-way repeated measures ANOVAs were performed on the amphetamine+O-2050 dataset to control for effects of O-2050 on baseline premature responding. Results showed significant overall effects for both amphetamine (*F*_1,12_ = 34.81, *p*<0.001) and O-2050 (*F*_2,24_ = 16.28, *p*<0.001) administration as well as an interaction effect between the two treatments (*F*_2,24_ = 14.16, *p*<0.001), suggesting that also pretreatment with O-2050 had stronger effects on premature responding combined with amphetamine compared to its own effects thereon. Significant treatment effects found on other behavioral parameters in the 5-CSRTT for the amphetamine and O-2050 experiment included accurate choice (Table 3; *F*_5,60_ = 8.96, *p*<0.001, ε = 0.67), with O-2050 dose-dependently restoring amphetamine-induced attentional deficits and improving attentional functioning when administered alone, and number of omissions (*F*_5,60_ = 13.53, *p*<0.001, ε = 0.47), with 1 mg/kg O-2050 increasing the number of omissions both when administered alone and in combination with amphetamine. Possibly related to the latter finding, and another indication of aspecific somatomotor effects, correct response latencies were also lengthened by the highest dose of O-2050 (*F*_5,60_ = 10.64, *p*<0.001). No significant treatment effects were observed for perseverative responses (*F*_5,60_ = 1.52, NS) and feeder latencies (*F*_5,60_ = 2.30, NS, ε = 0.25).

The effects of amphetamine alone and in combination with O-2050 were also tested in the DRT (Fig. 2c,d). One animal had to be excluded from the analysis in this experiment, due to a high omission rate (>70% omissions in choice trials in each delay block) on several test days. Again, similar to what was observed for SR141716A, O-2050 did not significantly alter impulsive choice by itself, but completely prevented amphetamine-induced reductions in impulsive choice (Treatment: *F*_5,60_ = 5.55, *p*<0.001; Treatment×Delay: *F*_20,240_ = 2.06, *p* = 0.006). Together, these data confirm that CB1 receptor activity modulates amphetamine-induced impulsive action as well as impulsive choice. Consequently, the previously observed effects of SR141716A on impulsivity were likely due to blockade of endocannabinoid-induced activation of the CB1 receptor rather than inverse agonism at this receptor.

### Effects of Δ9-THC on impulsivity in the 5-CSRTT and DRT

**Figure 3:**
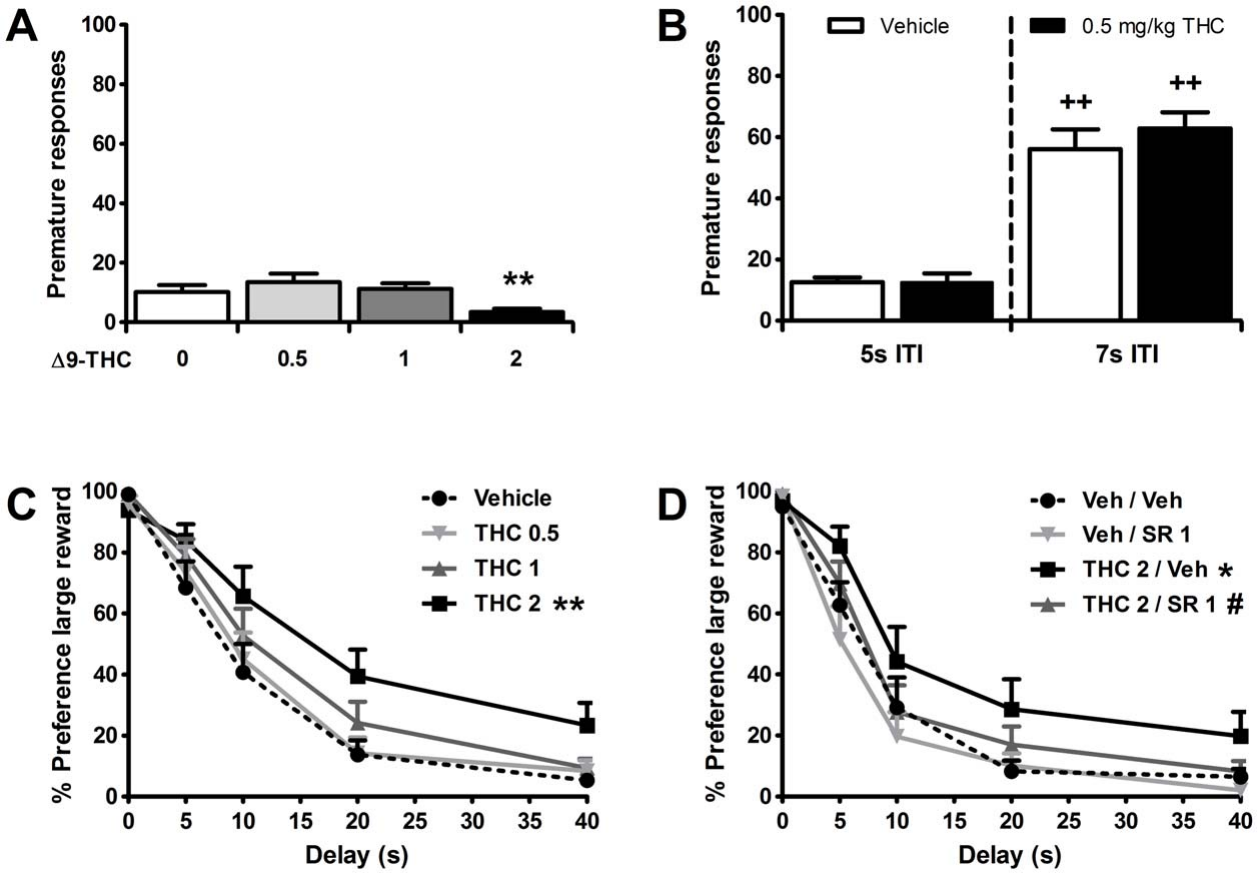
The CB receptor agonist Δ9-Tetrahydrocannabinol does not affect inhibitory control, but reduces impulsive choice. Effects of acute administration of Δ9-Tetrahydrocannabinol (THC) on the mean (± SEM) number of premature responses made in the 5-CSRTT (a,b) and effects of THC, SR141716A (SR), and their combination on the percentage preference for the larger, delayed reinforcer in the DRT (c,d). ITI: intertrial interval. In total *n* = 13−14 animals were included in the analyses. Drug doses are expressed as mg/kg. ^*^ *p*<0.05 and ^**^ *p*<0.005 versus Vehicle or Vehicle-Vehicle; ^#^ *p*<0.05 compared to THC-Vehicle, ^++^p<0.005 vs respective ITI 5 s control.

The aforementioned experiments with SR141716A and O-2050 indicate that CB1 receptor activation by endogenous ligands induces impulsive action as measured in the 5-CSRTT, while reducing impulsive choice in the DRT. Therefore, we next tested the effects of direct, agonist-induced CB1 receptor activation on impulsive behavior in rats. Δ9-THC was used as an exogenous, non-selective CB1 receptor agonist (*in vitro* Ki 5.1 and 3.1 nM for CB1 and CB2 receptors, respectively [59]), since this compound is regularly used in clinical studies and has previously been shown to acutely affect impulsivity in healthy volunteers [17], [18]. In the 5-CSRTT, one animal was excluded from the analyses due to a high number of omissions (>40) made under vehicle conditions. Results showed that acute administration of Δ9-THC affected premature responding (*F*_3,36_ = 7.60, *p*<0.001), with post-hoc tests revealing a significant reduction in impulsive responding following 2 mg/kg of Δ9-THC (Fig. 3a). However, as can be seen in Table 4, the same dose of Δ9-THC also significantly elevated correct response latencies (*F*_3,36_ = 12.36, *p* = 0.001, ε = 0.48) and number of omissions (*F*_3,36_ = 23.47, *p*<0.001, ε = 0.45). Hence, the effects on premature responding probably reflected general disturbance of task performance rather than a specific reduction in impulsive behavior. Other behavioral parameters in the 5-CSRTT were not affected by Δ9-THC (accurate choice: *F*_3,36_ = 1.38, NS; perseverative responding: *F*_3,36_ = 2.39, *p*<0.1; feeder latency: *F*_3,36_ = 2.06, NS, ε = 0.52).

**Table 4:** Effects of the CB1 receptor agonist Δ9-Tetrahydrocannabinol (THC) on measures of attentional function, compulsivity, and motivation in the 5-CSRTT.

Treatment (mg/kg)	Accuracy (%)	Perseverative responses	Correct response latency (ms)	Omissions	Feeder latency (ms)
**Vehicle<\emph>**	81.3±1.7	8.2±1.2	653±16	12.9±2.3	2091±249
**THC 0.5<\emph>**	83.9±1.4	4.9±1.3	698±18	11.6±1.8	1749±151
**THC 1<\emph>**	82.3±1.6	9.7±2.6	736±21	18.6±2.7	2327±355
**THC 2<\emph>**	85.6±2.8	4.2±1.3	929±62**	46.4±6.4**	3083±802

In total n = 13 animals were included in the analyses and data depict mean±SEM.

**p<0.005 versus Vehicle.

As we observed that the effects of both SR141716A and O-2050 on inhibitory control were particularly pronounced under lengthened ITI durations, the effects of a low dose (0.5 mg/kg) of Δ9-THC were next tested under these conditions on four additional test days. The same group of rats that was used for the initial dose-response curve of Δ9-THC was again tested, and one animal (the same one as before) was excluded from the analyses due to high omission rates under vehicle conditions. As in the SR141716A experiment (Fig. 1b), lengthening the ITI duration robustly increased premature responding in this cohort of rats (Fig. 3b; Treatment: *F*_1,12_ = 1.23, NS; ITI: *F*_1,12_ = 154.55, *p*<0.001; Treatment×ITI: *F*_1,12_ = 0.86, NS). Additionally, and somewhat contrasting the results with SR141716A (Table 2), lengthening the ITI duration this time also affected accuracy, the number of omissions made, and perserverative responding whilst not affecting correct response and feeder latencies (Table 5; accurate choice, Treatment: *F*_1,12_ = 0.10, NS; ITI: *F*_1,12_ = 18.27, *p* = 0.001; Treatment×ITI: *F*_1,12_ = 0.74, NS; perseverative responding, Treatment: *F*_1,12_ = 0.76, NS; ITI: *F*_1,12_ = 10.33, *p* = 0.007; Treatment×ITI: *F*_1,12_ = 2.43, NS; correct response latency, Treatment: *F*_1,12_ = 0.12, NS; ITI: *F*_1,12_ = 0.19, NS; Treatment×ITI: *F*_1,12_ = 0.08, NS; omissions, Treatment: *F*_1,12_ = 3.25, *p*<0.1; ITI: *F*_1,12_ = 9.37, *p* = 0.001; Treatment×ITI: *F*_1,12_ = 0.25, NS; feeder latency, Treatment: *F*_1,12_ = 1.17, NS; ITI: *F*_1,12_ = 0.61, NS; Treatment×ITI: *F*_1,12_ = 0.20, NS). Importantly however, under these experimental conditions Δ9-THC did not significantly affect premature responding, nor any other behavioral parameter in the 5-CSRTT.

**Table 5:** Effects of the CB1 receptor agonist Δ9-Tetrahydrocannabinol (THC) on measures of attentional function, compulsivity, and motivation in the 5-CSRTT under conditions of normal or lengthened intertrial interval (ITI).

Treatment (mg/kg)	ITI (s)	Accuracy (%)	Perseverative responses	Correct response latency (ms)	Omissions	Feeder latency (ms)
**Vehicle<\emph>**	5	82.3±1.7	5.3±1.8	629±16	9.3±2.2	1664±114
**THC 0.5<\emph>**	5	84.3±2.3	7.6±1.5	637±20	11.2±1.4	1757±155
**Vehicle<\emph>**	7	75.4±2.5++	2.6±0.4+	625±17	14.3±2.2++	1467±47
**THC 0.5<\emph>**	7	74.3±2.9++	1.8±0.6+	626±26	18.5±4.5++	1690±253

In total n = 13 animals were included in the analyses and data depict mean±SEM.

+ *p*<0.05.

++ *p*<0.005 compared to respective ITI = 5 s control.

Finally, the effects of Δ9-THC were tested in the DRT. As can be seen in Figure 3c, acute challenges with Δ9-THC dose-dependently reduced impulsive choice, with the highest dose (2 mg/kg) being significantly different from vehicle (Treatment: *F*_3,39_ = 9.85, *p*<0.001; Treatment×Delay: *F*_12,156_ = 2.30, *p* = 0.01). To verify that the observed Δ9-THC effect was CB1 receptor-mediated, effects of 2 mg/kg Δ9-THC alone and in combination with 1 mg/kg SR141716A were tested next in the same group of rats. One animal needed to be excluded from the analyses due to a high omission rate (>80% of choice trials per delay block) under Δ9-THC alone conditions. Results confirmed that the Δ9-THC-induced reduction in impulsive choice was mediated by the CB1 receptor, as it was completely abolished by pretreatment with SR141716A (Treatment: *F*_3,36_ = 6.16, *p* = 0.009, ε = 0.61; Treatment×Delay: *F*_12,144_ = 1.29, NS, ε = 0.77). Thus, it appears that although (endocannabinoid-induced) CB1 receptor activation modulates the opposite effects of amphetamine on impulsive action and impulsive choice, direct stimulation of the receptor by administration of an exogenous agonist only affects impulsive choice.

## Discussion

This study provides evidence for an important role of the cannabinoid CB1 receptor in modulating impulsive action as well as impulsive choice and the effects of the psychostimulant drug amphetamine thereon (Table 6). Previous studies showed that CB1 receptor antagonists with inverse agonistic properties increase baseline inhibitory control in the 5-CSRTT [20], [21]. The current study extends these findings by showing that amphetamine-induced decreases in inhibitory control, at least as measured in the 5-CSRTT, could be attenuated with the CB1 receptor antagonist/inverse agonist SR141716A. Moreover, SR141716A fully prevented the ameliorating effects of amphetamine on a different form of impulsivity, that is impulsive choice as measured in the DRT, while not affecting baseline behavior in the latter task. Importantly, in both behavioral paradigms, the effects of SR141716A were mimicked by the neutral CB1 receptor antagonist O-2050 [49], [57], [58]. The current results are therefore not compound-specific and can unlikely be attributed to inverse agonism at the CB1 receptor [56], but rather reflect the effects of blockade of CB1 receptor activation by endogenous cannabinoids. Furthermore, we found that, at least under the current baseline discounting curves with vehicle, direct CB1 receptor activation by administration of the CB receptor agonist Δ9-THC reduced impulsive choice without affecting impulsive action.

**Table 6:** Overview of the effects of CB1 receptor (ant)agonists on impulsive behavior found in this study.

	CB1 receptor antagonist		CB1 receptor agonist
	SR141716A	O-2050 (neutral)	Δ9-THC
**Impulsive action (5-CSRTT)**			
**Baseline (ITI = 5 s)**	↔*	↓	↔
**Baseline (ITI = 7 s)**	↓	↓	↔
**Amphetamine-induced (↑)**	↓	↓	N.D.
**Impulsive choice (DRT)**			
**Baseline**	↔	↔	↓
**Amphetamine-induced (↓)**	↑	↑	N.D.

Arrows indicate the direction of the effects of CB1 receptor (ant)agonists on impulsivity relative to baseline or amphetamine-induced levels of impulsivity, whereby amphetamine alone increases and decreases impulsive action and choice as compared to baseline, respectively.

*SR141716A has under these conditions previously been found to reduce impulsivity in the 5-CSRTT (Pattij et al. 2007a). N.D. not determined.

### CB1 receptor activity modulates amphetamine-induced impulsive action as well as impulsive choice

Psychostimulant drugs such as amphetamine are leading prescription drugs to treat ADHD and maladaptive display of impulsivity [26], [27]. The acute effects of amphetamine on inhibitory control [28]–[34] and impulsive choice [34]–[37] have been well characterized in both humans and rodents, and are known to depend on amphetamine's ability to robustly enhance mesocorticolimbic DA transmission [28], [29], [31]–[33], [36], [38], [39]. However, other neurotransmitter systems including the endogenous opioid and 5-HT systems have also been shown to regulate certain aspects of amphetamine-induced impulsivity [34], [38], [39]. Here, it was found that blocking the CB1 receptor with either SR141716A or O-2050 alleviated amphetamine-induced inhibitory control deficits and completely abolished amphetamine-induced decrements in impulsive choice, indicating that the endocannabinoid system plays a critical role in the opposite effects of amphetamine on impulsive action and impulsive choice. In addition, these findings add to previous data pointing towards a modulatory role for CB1 receptors in amphetamine's effects on other behaviors including locomotor activity, reward and motivation, and relapse to drug seeking [42]–[47].

Exactly how the endocannabinoid system modulates amphetamine-induced behaviors remains as yet largely unknown. For instance, data on the acute effects of amphetamine and other psychostimulants on endocannabinoid levels in the brain is scarcely available and rather inconclusive [40]–[42]. Since it is well known that particularly mesocorticolimbic DA projections critically regulate impulsive action and choice [28], [29], [31]–[33], [36], [38], [39], it is conceivable that CB1 receptor activity regulates (the effects of amphetamine on) impulsive action and impulsive choice by modulating mesocorticolimbic DA release. There is ample evidence that CB1 receptor activity can indirectly modulate DA release into brain areas such as the nucleus accumbens and medial prefrontal cortex [45], [60]–[63]. Similarly, there is evidence that DA receptor activation can activate the endocannabinoid system [41], [64], [65]. Together, these findings suggest that interactions between the DA and endocannabinoid systems, although being rather complex [65], may be critical in regulating different aspects of impulsive behavior. However, since CB1 receptor activity is capable of modulating release of virtually all other neurotransmitters [12], [13], it cannot be ruled out that indirect effects of CB1 receptor (ant)agonists on other neurotransmitter systems were responsible for the observed effects on (amphetamine-induced) impulsivity. For instance, CB1 and µ-opioid receptors closely interact, and even the existence of CB1/µ-opioid receptor heterodimers in certain brain regions has been suggested [58], [66]–[69]. Interestingly, it was recently shown that µ-opioid receptors in the nucleus accumbens shell subregion are critically involved in regulating amphetamine-induced changes in inhibitory control, but not impulsive choice [34]. This could implicate that CB1 and µ-opioid receptors are located in one neuronal population regulating inhibitory control, while being located in distinct neuronal populations regulating impulsive choice. Accordingly, we have recently observed that pretreatment with SR141716A prevented the reduction in inhibitory control, but not the increase in impulsive choice induced by the µ-opioid receptor agonist morphine (unpublished data). Together, our findings that CB1 receptor activity is differentially involved in modulating impulsive action as measured in the 5-CSRTT versus impulsive choice as measured in the DRT provide further evidence for a fractionation of impulsive behavior at the behavioral and neurochemical level [3], [4], [55], [70].

### CB1 receptor modulation of impulsive action

It should be noted that both SR141716A and O-2050 by themselves did not affect premature responding in the 5-CSRTT experiments with amphetamine. These findings are likely to reflect a floor effect, i.e. the low baseline level of premature responding presumably masked the enhancing effects of both compounds on inhibitory control. Similarly, SR141716A did not affect premature responding in a lateralized reaction time task, in which well-trained animals make very few premature responses under baseline conditions [71]. Moreover, when in the current study task demands were changed by lengthening the intertrial interval to increase the number of premature responses made, both SR141716A and O-2050 reduced premature responding. These findings clearly demonstrate that an endocannabinoid tone underlies this behavioral response. In fact, the above described observations combined with the current finding that CB1 receptor antagonists alleviated the high levels of impulsive responding induced by amphetamine suggest that the effects of CB1 receptor antagonists on inhibitory control are rate-dependent. Consequently, reducing endocannabinoid transmission may only enhance inhibitory control processes when the level of impulsive action is sufficiently high, independent of whether impulsive action was evoked pharmacologically or procedurally. Such a rate-dependent behavioral profile would aid the clinical interest in compounds that reduce endocannabinoid transmission, as specifically cohorts of patients suffering from high levels of impulsivity, such as ADHD patients and drug addicts, may then be expected to benefit from this type of treatment. Importantly, lower doses of SR141716A (1 mg/kg) and O-2050 (0.3 mg/kg) that already reduced (amphetamine-induced) premature responding did generally not affect any other behavioral parameter in the 5-CSRTT, except for a slight enhancement of accuracy by the lowest dose of O-2050, an interesting effect that was previously also found with 0.3 mg/kg SR141716A [21]. Parameters not affected by lower doses of both CB1 receptor antagonists include those that can be interpreted as indicators of somatomotor activity and food motivation, two behavioral aspects that are well known to be influenced by CB1 receptor activity [72], [73]. Particularly drug effects on food motivation could have confounded the interpretation of the data given the known positive relationship between CB1 receptor activity and appetite [72] and the positive correlation between food motivation and premature responding in the 5-CSRTT that has been reported [74]–[76]. However, although this hypothesis might explain the ameliorative effects of SR141716A and O-2050 on premature responding, it does not fit with the null effects of the CB receptor agonists Δ9-THC (current study) and WIN55,212–2 [21] on this type of impulsive behavior. Moreover, reduced food motivation has repeatedly been shown to result in increased correct response latencies, omission rates, and to a lesser extent, feeder latencies in the 5-CSRTT [74]–[76]. In the current study, correct response latencies and omission rate were only increased by doses of SR141716A and O-2050 that were higher than those required to reduce premature responding. In addition, feeder latencies were not affected by any drug treatment indicating that the effects of both CB1 receptor antagonists were probably unrelated to their anorectic properties. Another putative confounding factor might relate to the implementation of a fixed ITI duration in the current study, as rats could have adapted a timing strategy to help them predict the onset of stimulus presentations, a strategy that might simultaneously decrease premature responding. Consequently, given that both psychostimulants and cannabinoids are known to affect timing behavior [14], [77], the observed drug effects on premature responding might have been a reflection of distorted timing abilities rather than altered impulsivity. However, the observation that both psychostimulants and CB receptor agonists result in an underestimation of time [78], [79] and only psychostimulant administration results in increased premature responding in the 5-CSRTT argues against such an explanation. In addition, it was recently found that the CB1 receptor antagonist SLV330 reduced premature responding in a version of the 5-CSRTT that incorporated ITI durations of variable length rendering stimulus presentation unpredictable in time [20]. Similarly, amphetamine has been shown to increase premature responding in a 5-CSRTT with variable ITI durations [80], although in this particular study premature responses remained unpunished hampering the interpretation of this parameter as a readout for inhibitory control. Moreover, others have reported a reduction in impulsivity following amphetamine administration under similar conditions [81]. Altogether, these findings do not support a major role for altered time perception in the drug-induced changes in impulsivity observed in the current study. Collectively, although non-specific behavioral effects of the drugs used in this study cannot be ruled out completely, such effects are unlikely to have fully accounted for the effects of these compounds on impulsivity.

Considering the apparent important role of CB1 receptor activity in (amphetamine-induced) inhibitory control deficits, it was somewhat surprising that the CB receptor agonist Δ9-THC did not affect premature responding in the 5-CSRTT. Similar results were previously obtained for another synthetic CB receptor agonist, WIN55,212–2 [21]. Collectively, these data suggest that CB1 receptors regulating inhibitory control may already be maximally activated, for instance, due to excessive task-induced release of endogenous cannabinoids, thereby occluding effects of exogenous CB receptor agonists on impulsivity. Alternatively, distinct populations of CB1 receptors in the brain may exert opposite effects on premature responding. In the latter scenario, stimulation of all CB1 receptors by systemic administration of an agonist would have no *net* effect. Future experiments employing intracranial infusion of CB1 receptor agonists as well as inhibitors of endocannabinoid synthesis and hydrolysis may shed more light on this issue. In particular, such experiments including intracranial infusion of CB1 receptor antagonists will aid elucidating the anatomical locus where CB1 receptors modulate impulsivity. Considering the critical role of the prefrontal cortex and nucleus accumbens in regulating this behavior [3], [4], [55] and the high abundance of CB1 receptors in these brain areas [10], [11], these brain areas are likely candidates. However, a role for CB1 receptors in brain areas such as the ventral tegmental area, dorsal raphe nucleus and locus coeruleus cannot be ruled out at this point. Importantly, CB1 receptors can modulate the efferent output of these brain regions, thereby controlling monoaminergic input to brain areas including the prefrontal cortex and nucleus accumbens [82]–[84].

### CB1 receptor modulation of impulsive choice

In contrast to the absence of an effect of Δ9-THC in the 5-CSRTT, Δ9-THC decreased impulsivity in the DRT, an effect that was antagonized by SR141716A, hence, was CB1 receptor-dependent. Together with the finding that CB1 receptor antagonists completely abolished amphetamine-induced decrements in impulsive choice without affecting baseline impulsive choice behavior, this suggests that although CB1 receptors are not involved in mediating baseline impulsive choice, targeted CB1 receptor activation could be used to alleviate problems with delay aversion. In contrast, acute Δ9-THC administration in healthy volunteers does not seem to affect impulsive choice in a DRT [17]. However, as discussed before [85], fundamental differences between the human and rodent version of the DRT may account for this discrepancy. The current findings with Δ9-THC in the DRT also contrast the previously observed lack of effect of WIN55,212-2 in this task [21]. This inconsistency may be related to the fact that both agonists have completely dissimilar chemical structures and consequently differ in e.g. efficacy and binding profile at the CB1 receptor [59], [86]. To clarify this issue, the effects of other CB1 receptor agonists should be tested in the DRT. Particularly the effects of exogenous administration of the endocannabinoid anandamide would be interesting in this respect, since anandamide resembles Δ9-THC in being a partial CB1 receptor agonist [87] and ADHD patients were found to have decreased anandamide degradation as compared to healthy control subjects [25].

### Concluding remarks

The current results indicate an important, though complex role for CB1 receptor activity in regulating impulsive actions and impulsive choice as well as the opposite effects of amphetamine on both aspects of impulsivity. These data extend previous findings on the role of CB1 receptors in impulsive behavior, including clinical findings linking polymorphisms in the CB1 receptor gene (CNR1 gene) to impulsivity [22] and the development of ADHD [23], [24]. Although the mechanism underlying CB1 receptor modulation of impulsivity still remains unknown, and the endogenous ligands involved elusive, the CB1 receptor may be an interesting novel target for pharmacotherapies to treat maladaptive impulsivity. According to the dual pathway model for the development of ADHD [88], [89], our data would then suggest that patients suffering from a primarily motivational, delay aversion subtype of ADHD may benefit from enhanced CB1 receptor activation, whereas on the other hand patients whose disorder is more related to poor inhibitory control may benefit from reduced CB1 receptor activity. Future studies will have to explore under which exact conditions CB1 receptor-targeted drugs can be helpful to treat maladaptive impulsivity.
